# Parental health belief model constructs associated with oral health behaviors, dental caries, and quality of life among preschool children in China: a cross-sectional study

**DOI:** 10.1186/s12903-024-05290-7

**Published:** 2024-12-18

**Authors:** Shu-Mei Liu, Yu-Meng Xin, Feng Wang, Pei-Chao Lin, Hsiao-Ling Huang

**Affiliations:** 1 Department of Preschool Education, Jing Hengyi College of Education, Zhejiang Philosophy and Social Science Laboratory for Research in Early Development and Childcare, Hangzhou Normal University, Hangzhou, China; 2 Department of Health Policy and Management, School of Public Health, Hangzhou Normal University, Hangzhou, China; 3 School of Nursing, College of Nursing, Kaohsiung Medical University, Kaohsiung, Taiwan; 4 Center for Long-Term Care Research, Kaohsiung Medical University, Kaohsiung, Taiwan; 5 Department of Medical Research, Kaohsiung Medical University Hospital, Kaohsiung Medical University, Kaohsiung, Taiwan; 6 Department of Oral Hygiene, College of Dental Medicine, Kaohsiung Medical University, 100 Shih-Chuan 1st Road, Kaohsiung City, 80708 Taiwan

**Keywords:** Health belief model, Oral health behaviors, Early childhood caries, Oral health related-quality of life, Preschool children

## Abstract

**Background:**

Early childhood caries (ECC) is a prevalent health problem that negatively affects both overall health and oral health–related quality of life (OHRQoL). This study investigated the association between health belief model (HBM) constructs and oral health behaviors, dental caries, and OHRQoL in preschool children in China.

**Methods:**

A total of 1562 preschool children aged 3 to 6 years were recruited from six public kindergartens in Hangzhou, Zhejiang Province, by using stratified cluster sampling. A questionnaire was administered to parents to collect information on demographics, HBM constructs, oral health behaviors, dental caries, and OHRQoL. Regression models were used to examine relationships between HBM constructs and the outcomes.

**Results:**

Parental perceived benefits [adjusted odds ratio (AOR) = 1.47 and 1.42], perceived barriers (AOR = 0.65 and 0.63), and oral health self-efficacy (AOR = 20.59 and 19.09) were associated with brushing teeth twice daily and brushing teeth with parental assistance. Perceived susceptibility (AOR = 6.62) and perceived severity (AOR = 0.49) were significantly associated with children’s ECC. Poorer oral health (β = 0.09), higher perceived susceptibility (β = 0.11), and greater perceived barriers (β = 0.30) were associated with lower OHRQoL. Brushing teeth twice daily (β = −0.19) and brushing teeth with parental assistance (β = −0.09) were associated with higher OHRQoL.

**Conclusions:**

Parental HBM constructs were significantly associated with oral health behaviors, ECC, and OHRQoL in preschool children. These findings indicate the importance of incorporating parental HBM constructs into health education programs to promote positive oral health behaviors, reduce the prevalence of caries, and enhance OHRQoL in preschoolers.

**Trial registration:**

Not applicable.

**Supplementary Information:**

The online version contains supplementary material available at 10.1186/s12903-024-05290-7.

## Introduction

Early childhood caries (ECC) is a major global oral health problem and the most prevalent chronic disease in children [1]. Approximately 514 million children globally have caries in their primary teeth [2]. In China, the prevalence of dental caries among children aged 3, 4, and 5 years reached 50.8%, 63.6%, and 71.9%, respectively, in 2018 [3]. These rates reflect increases of 10.6%, 9.2%, and 7.8% over the decade preceding 2018, indicating a concerning rise in dental caries among young children in China [4]. Children with untreated ECC have poorer oral health behaviors and lower oral health-related quality of life (OHRQoL) than do their counterparts without ECC [5, 6]. ECC can result in pain, infection, and difficulties in eating and speaking. In addition, ECC can adversely affect the health of permanent teeth and have psychological effects, such as low self-esteem and poor social communication, further affecting OHRQoL [5, 7]. ECC imposes a substantial burden on parents, requiring them to dedicate time and resources to their child’s dental care [8].

Parents play a crucial role in shaping their child’s oral health status and behaviors [9, 10]. However, most studies have focused on the relationship of caries with sociodemographic factors, feeding behaviors, and various psychosocial factors in parents, such as attitude, knowledge, self-efficacy, and beliefs [11–13]. Few studies have specifically investigated the effect of parental psychosocial factors on children’s oral health behaviors and quality of life, especially in the context of ECC determinants and their effects on quality of life. Although several studies have reported that dental caries affect quality of life and that parental perceptions affect the oral health behaviors of preschool children [5, 8, 14], the extent to which parental psychosocial factors affect preschool children’s dental caries, oral health behaviors, and quality of life remains unclear.

The health belief model (HBM), originally developed by Becker in 1974 and later refined by Rostenstock in 1990 [15], is based on the theory that an individual’s readiness to change their health behaviors is primarily influenced by their perceptions of health. The HBM has been widely used to examine health beliefs associated with preventive oral health behaviors [16, 17]. According to this model, individual beliefs regarding health and health conditions substantially affect health-related behaviors. The HBM has six main components: perceived severity, perceived susceptibility, perceived benefits, perceived barriers, self-efficacy, and behavior. The first four constructs—perceived severity, perceived susceptibility, perceived benefits, and perceived barriers—were the foundational elements of this theory when it was initially developed. Self-efficacy was later incorporated into the HBM to enhance its original framework and improve its predictive capability [15]. Maternal self-efficacy and oral health beliefs are correlated with both maternal and children’s behavior related to oral health [11, 12]. Parents with higher self-efficacy, greater perceived benefits, higher awareness of severity, and fewer perceived barriers are more likely to implement the oral health practices recommended for preschool children. Furthermore, better oral health outcomes in children are associated with higher levels of parental self-efficacy and lower levels of perceived severity and barriers [18, 19]. HBM-based interventions can empower parents to promote appropriate oral health behaviors and improve their children’s oral health [20]. However, the HBM has not adequately addressed the associations of self-efficacy and oral health beliefs with OHRQoL. Negative emotions related to oral health, such as fear and anxiety, may affect perceived susceptibility and barriers [21]. Thus, the relationships between HBM constructs, oral health behaviors, dental caries, and OHRQoL in preschool children should be investigated.

Determining the propositional structures of the HBM could facilitate the design of theory-based interventions to improve OHRQoL and prevent dental caries. In this study, we used the HBM as a theoretical framework to identify factors affecting oral health behaviors, dental caries, and OHRQoL in preschool Chinese children. A comprehensive understanding of these factors could support the development and implementation of personalized interventions for promoting positive behavioral changes related to preschool children’s oral health.

## Methods

### Study design

This study used an analytical cross-sectional study design in accordance with the STROBE (Strengthening the Reporting of Observational Studies in Epidemiology) guidelines [22]. The parents of preschool children were recruited from licensed kindergartens in Hangzhou, Zhejiang. This study was conducted over a period of 2 months from October to November 2022. Clinical Trial Registration number: not applicable.

### Participants

A stratified cluster sampling method was used to select the study sample. In the first semester of the 2022–2023 academic year, 392,700 preschool children were enrolled in 1073 licensed kindergartens according to the official website of Hangzhou Education Committee [23]. Three districts were randomly selected from the 10 municipal districts of Hangzhou. Participants were allocated proportionally on the basis of the number of children in each district’s kindergartens. In these districts, six kindergartens were randomly chosen from the list of licensed kindergartens, and three to five classes were selected from each kindergarten for the study.

The sample size for logistic regression was calculated using G*Power 3.1.9.4 software (HHU, Dusseldorf, Germany). The appropriate sample number was estimated to be 948, assuming a statistical power of 0.8, two-tailed α of 0.05, odds ratio of 1.3 [24], and proportion of 0.14 in the ECC group [25]; the independent variable was assumed to follow a normal distribution. To account for an estimated 20% loss rate, a final sample size of 1,137 participants was recommended.

The research protocol was approved by the Research Ethics Committee of Jing Hengyi College of Education at Hangzhou Normal University Institutional Review Board (IRB No. 2022013). From October to November 2022, parents were invited to complete an online questionnaire through the Wen Juanxing app (the Chinese equivalent of Survey Monkey, https://www.wjx.cn); completing it took 15 to 20 min. The participants were informed of their right to withdraw from the survey at any time. Incomplete questionnaires were excluded from the analysis. Out of the initial pool of 1,700 eligible participants, 1,562 completed questionnaires were included in the final analysis.

### Measures

The self-administered questionnaire was developed on the basis of previously designed measures, namely the Early Childhood Oral Health Impact Scale (ECOHIS) [26], the 4th National Oral Health Survey of China [3], the Early Childhood Caries Perceptions Scale (ECCPS) [27], and the Parental Oral Health Self-Efficacy Scale [28]. A panel of six external experts in health behaviors and oral health reviewed the questionnaire to confirm its relevance and accuracy. In addition, the questionnaire was pretested with 30 parents to evaluate the suitability and reliability of its items. Items with lower internal consistency were revised to enhance clarity and comprehension. The pretest results indicated that the overall internal consistency of the questionnaire was adequate for use in a large-scale survey (Cronbach’s α > 0.7).

### Dependent variables

#### Child’s OHRQoL

Child’s OHRQoL was examined using the ECOHIS. A Chinese version of the ECOHIS was developed, and this version was reported to demonstrate acceptable validity and reliability (Cronbach’s α = 0.91) [26]. The ECOHIS is based on parental ratings of 13 items, divided into two subscales: The Child Impact Section (CIS) with 9 items and the Family Impact Section (FIS) with 4 items. The CIS has four subdomains: child symptoms, child function, child psychology, and child self-image and social interaction. The FIS consists of two domains: parental distress and family function. Each question is rated on a 5-point Likert scale, indicating the frequency of events in the child’s life. The response options range from “never” (scored as 1) to “always” (scored as 5; Table S-1). The overall ECOHIS score is calculated by summing the responses to all 13 items, with a higher score indicating a stronger impact of oral health problems and a lower level of OHRQoL. In the current study, the ECOHIS demonstrated favorable internal consistency, with a Cronbach’s α coefficient of 0.97.

#### Child’s dental caries

A child’s dental caries were assessed through parental reports by using the following question: “How many dental caries (decayed, missing, or filled teeth) has your child had so far?” The response options were “zero” (scored as 0), “one” (scored as 1), “two” (scored as 2), “three” (scored as 3), and “four or more” (scored as 4). If parents selected “zero,” it was coded to indicate that the child had no dental caries. Any other response was coded to indicate that the child had dental caries.

#### Child’s oral health behaviors

A child’s oral health behaviors were assessed using four items: brushing teeth twice daily, brushing teeth with parental assistance, daily consumption of sweet foods, and recent dental examination. The parent participants were asked the following questions: (1) “In the past month, did your child brush his or her teeth every morning and evening?” (2) “In the past month, did you help your child brush his or her teeth?” (3) “In the past month, did your child consume sweet foods every day?” (4) “In the past 6 months, did your child visit the dentist for a dental examination?” Parents responded with either “yes” or “no” for each item (Table S-2).

### Independent variables

#### Sociodemographic variables

The sociodemographic variables were the child’s sex (boy vs. girl), child’s age (3 < age ≤ 4, 4 < age ≤ 5, and 5 < age ≤ 6 years), parental identity (mother vs. father), and parental educational qualification (high school or lower, college, and bachelor’s degree or higher).

#### Parental oral health knowledge

Parental oral health knowledge was evaluated using 14 items across five dimensions: (1) dental knowledge (three items, e.g., “Deciduous teeth are replaced later and have no impact on permanent teeth”), (2) dietary habits (three items; e.g., “Providing children with excessive sweet foods or carbonated drinks often has a detrimental impact on their teeth”), (3) oral hygiene tools (three items, e.g., “Children’s toothbrushes should be replaced every six months”), (4) tooth brushing routines (three items; e.g., “It is challenging for young children to effectively brush their teeth, and parental assistance and supervision are needed”), and (5) dental visits (two items; e.g., “Sealing cavities and grooves in the teeth of preschool children can prevent tooth decay”). The participants responded to each statement with “correct,” “wrong,” or “I don’t know” (Table S-3). Correct responses scored 1 point, whereas the other two responses scored 0. A higher total score indicated a greater level of oral health knowledge. The Kuder–Richardson Cronbach’s α for the parental oral health knowledge scale was 0.80.

#### Parental oral health beliefs

Parental oral health beliefs were evaluated using the ECCPS, which was developed on the basis of the constructs of the HBM [27]. The ECCPS was used to assess health beliefs associated with ECC prevention among the primary caregivers of children aged under 5 years. The ECCPS consists of 20 items and has four subscales (Table S-4):

1. Perceived susceptibility reflects parents’ perceptions of the likelihood of their child developing dental caries and is measured using four items. For example, one item is “It is likely that my child will get tooth decay.”
2. Perceived severity indicates parents’ perceptions of the seriousness of dental caries and their consequences and is measured using six items. An example item is “Toothache can keep a child from sleeping through the night.”
3. Perceived benefits represent parents’ understanding of the positive health outcomes associated with their child practicing oral health care and are assessed using five items. A sample item is “Habitually child’s oral health cleaning will help my child avoid cavities.”
4. Perceived barriers highlight parents’ perceptions of the costs or obstacles to practicing oral health care for their child and are measured using five items. For instance, one item is “A child’s toothbrush is too expensive for me to provide to my child.”

Each item is rated on a 5-point Likert scale ranging from 1 (strongly disagree) to 5 (strongly agree). The Cronbach’s α for the ECCPS was 0.89, indicating good internal consistency.

#### Parental oral health self-efficacy

Parental oral health self-efficacy was examined using an instrument developed by Kakudate [28]. The participants were asked to respond to 16 questions on oral health self-efficacy across three dimensions: (1) self-efficacy for brushing teeth (six items; e.g., “I am confident in finishing brushing my child’s teeth even if he or she feels sleepy”); (2) self-efficacy for dietary habits (six items; e.g., “I am confident I can resist buying snacks at the supermarket even if my child asks me to”); and (3) self-efficacy for dentist consultations (four items; e.g., “I am confident I can continue to take my child to a dentist for regular checkups after treatment is finished”). Responses were recorded on a 5-point Likert scale ranging from 1 (strongly disagree) to 5 (strongly agree; Table S-5). A higher score indicated a greater level of parental oral health self-efficacy. The Cronbach’s α for the Parental Oral Health Self-Efficacy Scale was 0.96, indicating excellent internal consistency.

### Data analysis

Statistical analysis was performed using SPSS software version 26.0 (IBM, Armonk, NY, USA). A *p* value of < 0.05 indicated statistical significance. Descriptive statistics were calculated for all variables. A multinomial logistic regression model was used to identify factors affecting preschool children’s oral health behaviors and dental caries while controlling for sociodemographic variables. In addition, multiple linear regression analysis was performed to identify factors associated with preschool children’s OHRQoL while controlling for sociodemographic variables.

## Results

### Demographic distribution for children and parents

A total of 1562 children aged 3 to 6 years were included—801 boys (51.3%) and 761 girls (48.7%). The fitness test results (goodness-of-fit test) revealed no significant difference in sex distribution between the population and the sample size (*χ²* = 0.16, *p* > 0.05). Of the parents who completed the questionnaire, 81.6% were mothers and 18.4% were fathers. In total, 913 of the enrolled children (58.5%) had dental caries. As listed in Tables 1, 45.5% of the parents had an undergraduate or graduate degree, 27.5% had a junior college education, and 19.4% had a high school or lower qualification.

**Table 1:** Demographic distributions of children and parents

Variables	*n*	%	Early childhood caries				χ²	*p*
			No		Yes			
			*n*	%	*n*	%		
Child’s gender							0.08	0.782
Boy	801	51.3	336	51.7	465	50.9		
Girl	761	48.1	313	48.3	448	49.1		
Child’s age							58.83	**< 0.001**
3 < age ≤ 4	387	24.8	164	25.2	335	36.6		
4 < age ≤ 5	676	43.3	223	34.3	164	17.9		
5 < age ≤ 6	499	31.9	262	40.4	414	45.4		
Parental identity							6.22	**0.013**
Mother	1274	81.6	510	78.5	764	83.6		
Father	288	18.4	139	21.5	169	16.4		
Parental educational qualification							7.08	**0.029**
High school or lower	328	19.4	129	19.8	199	21.7		
College	465	27.5	175	26.9	290	31.8		
Bachelor’s degree or higher	769	45.5	345	53.2	424	46.5		
Child’s Oral health behavior								
Brushing teeth twice daily							0.12	0.729
No	217	13.8	93	14.3	124	13.5		
Yes	1345	86.2	556	85.7	789	86.5		
Brushing teeth with parental assistance							0.16	0.640
No	295	18.8	119	18.3	176	19.2		
Yes	1267	81.2	530	81.7	737	80.8		
Sweets consumption daily							4.95	0.026
No	1303	83.4	558	85.9	745	81.5		
Yes	239	16.6	91	14.1	168	18.5		
Recent dental examination							10.90	**0.001**
No	771	49.3	353	54.3	418	45.7		
Yes	791	50.7	296	45.7	495	54.3		
Parental oral health knowledge	11.76	1.74	11.64	1.70	11.86	1.73	-2.48	**0.013**
Perceived susceptibility	13.04	3.34	11.06	2.95	14.45	2.86	-22.83	**< 0.001**
Perceived severity	22.42	3.64	22.31	3.68	22.50	3.62	-1.03	0.302
Perceived benefits	19.56	3.22	19.47	3.25	19.62	3.22	-0.91	0.365
Perceived barriers	10.44	3.22	10.53	3.25	10.38	3.22	0.91	0.365
Parental oral health self-efficacy	57.53	8.35	57.54	7.99	57.22	8.60	0.04	0.967
Oral health-related quality of life	20.29	9.62	18.20	8.78	21.72	9.91	-7.50	**< 0.001**
Child impact	13.29	6.72	11.99	6.09	14.21	7.00	-6.70	**< 0.001**
Family impact	7.00	3.61	6.21	3.33	7.55	3.70	-7.48	**< 0.001**

*Note* Those who did not answer were not included, *n* = 1562

The findings revealed that the parents had a high level of oral health knowledge (mean: 0.84, score range: 0–1). In addition, the parents exhibited high levels of perceived susceptibility, perceived severity, and perceived benefits (means: 3.26, 3.74, and 3.91, respectively; score range: 1–5), whereas perceived barriers were at a moderate level (mean: 2.36, score range: 1–5). The parent participants exhibited good oral health self-efficacy (mean: 3.60, score range: 1–5). The total mean score for OHRQoL was 1.56 ± 0.74, with child impact at 1.48 ± 0.74 and parent impact at 1.75 ± 0.90 (score range: 1–5; Table 2). Regarding the children’s oral health behaviors, over 80% of parents reported good practices, such as brushing teeth twice daily (86.2%), brushing teeth with parental assistance (81.2%), and avoiding daily consumption of sweet foods (83.4%). However, only 50.7% of the parents reported that their child had undergone an oral health examination in the preceding 6 months (Table 1).

**Table 2:** Descriptive statistics of crucial variables and reliability

Variables	Items	Score	Mean	SD	Cronbach’s alpha
Oral health knowledge	14	0–1	0.84	0.13	0.796
Dental knowledge	3	0–1	0.88	0.22	
Dietary habits	3	0–1	0.96	0.13	
Oral hygiene tools	3	0–1	0.73	0.23	
Teeth brushing routines	3	0–1	0.77	0.19	
Dental visit	2	0–1	0.90	0.25	
Oral health beliefs					
Perceived susceptibility	4	1–5	3.26	0.84	0.720
Perceived severity	6	1–5	3.74	0.61	0.889
Perceived benefit	5	1–5	3.91	0.64	0.909
Perceived barriers	5	1–5	2.36	0.72	0.853
Oral health self-efficacy	16	1–5	3.60	0.52	0.969
Self-efficacy for brushing teeth	6	1–5	3.55	0.72	
Self-efficacy for dietary habit	6	1–5	3.56	0.53	
Self-efficacy for dentist consultations	4	1–5	3.72	0.67	
Oral health-related quality of life	13	1–5	1.56	0.74	0.976
Child impact	9	1–5	1.48	0.75	
Family impact	4	1–5	1.75	0.90	

*Note* Those who did not answer were not included, *n* = 1562. SD: standard deviation

### Factors related to children’s oral health behaviors

We analyzed factors associated with children’s oral health behaviors while controlling for sociodemographic variables (Table 3). The factors affecting the behavior of brushing teeth twice daily were parents’ perceived benefits [adjusted odds ratio (AOR) = 1.47, 95% confidence interval [CI]: 1.06–2.05, *p* = 0.023], perceived barriers (AOR = 0.65, 95% CI: 0.49–0.87, *p* = 0.004), and oral health self-efficacy (AOR = 20.59, 95% CI: 12.62–33.60, *p* < 0.001). The significant factors affecting the behavior of brushing teeth with parental assistance were parents’ perceived benefits (AOR = 1.42, 95% CI: 1.05–1.93, *p* = 0.025), perceived barriers (AOR = 0.63, 95% CI: 0.49–0.82, *p* = 0.001), and oral health self-efficacy (AOR = 19.09, 95% CI: 12.31–29.60, *p* < 0.001). In addition, the factors associated with the behavior of consuming sweet foods daily were perceived susceptibility (AOR = 1.67, 95% CI: 1.38–2.04, *p* < 0.001) and oral health self-efficacy (AOR = 0.36, 95% CI: 0.26–0.48, *p* < 0.001). Finally, the factors related to having dental examinations in the past six months were perceived susceptibility (AOR = 1.38, 95% CI: 1.18–1.62, *p* < 0.001) and oral health self-efficacy (AOR = 11.73, 95% CI: 8.45–16.28, *p* < 0.001).

**Table 3:** Factors related to child’s oral health behaviors

Variables	Brushing teeth daily				Brushing teeth with parental assistance				Sweet food consumption daily				Dental examination in the past six months			
	AOR	95%CI		*p*	AOR	95%CI		*p*	AOR	95%CI		*p*	AOR	95%CI		*p*
Child’s gender																
Boy	Ref.				Ref.				Ref.				Ref.			
Girl	1.39	0.98	1.95	0.062	0.88	0.65	1.19	0.391	1.11	0.84	1.46	0.480	1.01	0.80	1.27	0.950
Child’s age																
3 < age ≤ 4	Ref.				Ref.				Ref.				Ref.			
4 < age ≤ 5	2.10	1.3	3.22	**0.001**	0.75	0.49	1.13	0.167	0.70	0.50	0.98	**0.034**	1.27	0.95	1.71	0.104
5 < age ≤ 6	1.62	1.04	2.51	**0.033**	0.47	0.31	0.73	**0.001**	0.51	0.35	0.74	**< 0.001**	1.26	0.93	1.72	0.142
Parental identity																
Father	Ref.															
Mother	0.72	0.47	1.10	0.126	0.76	0.52	1.11	0.159	1.20	0.84	1.72	0.314	0.89	0.66	1.21	0.446
Parental educational qualification																
High school or lower	Ref.															
College	0.99	0.61	1.61	0.978	1.03	0.68	1.56	0.898	1.10	0.74	1.64	0.631	0.95	0.69	1.32	0.759
Bachelor’s degree or higher	0.85	0.54	1.34	0.494	1.10	0.74	1.64	0.631	1.00	0.68	1.45	0.980	1.17	0.86	1.59	0.333
Parental oral health knowledge	2.65	0.70	10.00	0.150	1.68	0.49	5.79	0.414	0.44	0.14	1.32	0.141	2.17	0.74	6.35	0.159
Perceived susceptibility	1.08	0.85	1.37	0.528	1.18	0.95	1.46	0.140	1.67	1.38	2.04	**< 0.001**	1.38	1.18	1.62	**< 0.001**
Perceived severity	1.14	0.79	1.66	0.489	1.28	0.91	1.79	0.160	1.21	0.88	1.64	0.240	0.82	0.63	1.07	0.139
Perceived benefits	1.47	1.06	2.05	**0.023**	1.42	1.05	1.93	**0.025**	1.25	0.95	1.65	0.114	1.22	0.97	1.54	0.094
Perceived barriers	0.65	0.49	0.87	**0.004**	0.63	0.49	0.82	**0.001**	0.87	0.71	1.08	0.197	0.89	0.74	1.07	0.212
Oral health self-efficacy	20.59	12.62	33.60	**< 0.001**	19.09	12.31	29.60	**< 0.001**	0.36	0.26	0.48	**< 0.001**	11.73	8.45	16.28	**< 0.001**

*Note* Those who did not answer were not included, *n* = 1562. AOR: adjusted odds ratio; Ref: reference

### Factors related to children’s dental caries and OHRQoL

The analysis revealed that higher parental perceived susceptibility (AOR = 6.62, 95% CI: 5.34–8.21, *p* < 0.001) and lower perceived severity (AOR = 0.49, 95% CI: 0.37–0.65, *p* < 0.001) were associated with children’s dental caries (Table 4). In the analysis of children’s OHRQoL, the factors associated with children’s OHRQoL were brushing teeth twice daily (β = −0.19, 95% CI: −0.52 to − 0.29, *p* < 0.001), brushing teeth with parental assistance (β = −0.09, 95% CI: −0.27 to − 0.06, *p* = 0.002), recent dental examination (β = 0.13, 95% CI: 0.12–0.26, *p* < 0.001), having dental caries (β = 0.09, 95% CI: 0.06–0.22, *p* = 0.001), perceived susceptibility (β = 0.11, 95% CI: 0.05–0.15, *p* < 0.001), and perceived barriers (β = 0.30, 95% CI: 0.26–0.35, *p* < 0.001; Table 5).

**Table 4:** Factors related to child’s early childhood caries

Variables	AOR	95%CI		*p*
Child’s gender				
Boy	reference			
Girl	1.05	0.82	1.34	0.699
Child’s age				
3 < age ≤ 4	reference			
4 < age ≤ 5	2.33	1.71	3.17	**< 0.001**
5 < age ≤ 6	3.09	2.20	4.32	**< 0.001**
Parental identity				
Father	reference			
Mother	0.65	0.47	0.89	**0.008**
Parental educational qualification				
High school or lower	reference			
College	1.12	0.79	1.60	0.521
Bachelor’s degree or higher	0.88	0.63	1.23	0.457
Parental oral health knowledge	1.49	0.52	4.23	0.457
Perceived susceptibility	6.62	5.34	8.21	**< 0.001**
Perceived severity	0.49	0.37	0.65	**< 0.001**
Perceived benefits	0.94	0.74	1.20	0.613
Perceived barriers	0.89	0.74	1.08	0.250
Child’s oral health behaviors				
Brushing teeth twice daily				
No	reference			
Yes	0.89	0.58	1.38	0.599
Brushing teeth with parental assistance				
No	reference			
Yes	0.83	0.55	1.24	0.361
Consumption of sweet foods daily				
No	reference			
Yes	1.12	0.79	1.58	0.523
Recent dental examination				
No	reference			
Yes	1.29	0.99	1.684	0.061

*Note* Those who did not answer were not included, *n* = 1562. AOR: adjusted odds ratio

**Table 5:** Factors related to oral health-related quality of life

Variables	*β*	95%CI		*p*
Child’s gender				
Boy	reference			
Girl	-0.01	-0.08	0.05	0.752
Child’s age				
3 < age ≤ 4	reference			
4 < age ≤ 5	0.04	-0.02	0.14	0.151
5 < age ≤ 6	0.09	0.05	0.23	**0.002**
Parental identity				
Father	reference			
Mother	0.08	0.07	0.24	**< 0.001**
Parental educational qualification				
High school or lower	reference			
College	0.04	-0.04	0.15	0.233
Bachelor’s degree or higher	0.05	-0.01	0.16	0.096
Parental oral health knowledge	-0.05	-0.54	0.01	0.058
Perceived susceptibility	0.11	0.05	0.15	**< 0.001**
Perceived severity	0.04	-0.03	0.11	0.224
Perceived benefits	0.01	-0.06	0.06	0.978
Perceived barriers	0.30	0.26	0.35	**< 0.001**
Child’s oral health behaviors				
Brushing teeth twice daily				
No	reference			
Yes	-0.19	-0.52	-0.29	**< 0.001**
Brushing teeth with parental assistance				
No	reference			
Yes	-0.09	-0.27	-0.06	**0.002**
Consumption of sweet foods daily				
No	reference			
Yes	0.02	-0.06	0.12	0.485
Recent dental examination				
No	reference			
Yes	0.13	0.12	0.26	**< 0.001**
Early childhood caries				
No	reference			
Yes	0.09	0.06	0.22	**0.001**

*Note* Those who did not answer were not included, *n* = 1562

## Discussion

The findings of the present study indicated that the constructs of the HBM were associated with children’s oral health behaviors, ECC, and OHRQoL. In particular, higher perceived susceptibility and lower perceived severity were significantly associated with ECC, whereas poorer oral health and higher perceived susceptibility and perceived barriers were associated with lower OHRQoL. Furthermore, brushing teeth twice daily and brushing with parental assistance were found to be correlated with higher OHRQoL. In addition, parents’ higher perceived benefits, lower perceived barriers, and greater self-efficacy were associated with better oral health behaviors in children, including regular teeth brushing and brushing with parental assistance. By contrast, parents’ higher perceived severity and lower self-efficacy were associated with poorer oral health behaviors in children, such as daily consumption of sweet foods.

The findings of the current study indicated that increased parental perceived susceptibility and perceived barriers were associated with decreased OHRQoL. A previous study reported that high perceived susceptibility and perceived barriers are crucial factors contributing to dental anxiety [21]. In this context, the present study further revealed a significant discrepancy in OHRQoL between children without dental caries and those with dental caries, supporting the findings of previous research on the relationship between dental caries and OHRQoL in preschoolers [14, 29]. In addition, our findings showed that positive oral health behaviors in children, such as regular teeth brushing and brushing with parental assistance, were associated with higher OHRQoL; this finding is consistent with those of other studies [30, 31]. However, we discovered that having a dental examination in the preceding 6 months was negatively associated with higher OHRQoL. A previous study demonstrated that early preventive dental visits were associated with lower prevalence of dental caries and untreated dental caries [32]. In our study, children with dental caries were more likely to have sought dental services for toothache in the preceding 6 months. Because the dental visit behavior of children was reported by their parents, it is possible that children with a higher prevalence of dental caries were more frequently taken for treatment, which may have contributed to the higher frequency of dental visits observed.

The present study found that higher parental perceived susceptibility and lower perceived severity were positively correlated with children’s dental caries. A previous study indicated that individuals with higher perceived susceptibility to oral disease were more likely to develop dental caries [21]. We inferred that parents with dental caries themselves may be more likely to perceive their children as susceptible to dental issues than are parents without dental caries. In addition, Hazavei et al. identified a correlation between perceived severity and dental caries in children aged 6 to 7 years in Hamadan [33], this finding is consistent with that of the present study. These results indicate the need for oral health educational intervention programs in preschools to enhance parents’ awareness of the risks associated with ECC. By increasing their understanding of susceptibility to and the severity of dental problems, such programs can ultimately improve their children’s oral health.

Our study revealed that parents with higher perceived benefits and lower perceived barriers were more likely to exhibit better oral health behaviors. This finding aligns with those of studies that have consistently linked perceived benefits, perceived barriers, and self-efficacy to positive oral health behaviors [34, 35]. Specifically, perceived benefits play a crucial role in promoting preventive behaviors [36]. Individuals with high perceived benefits and low perceived barriers are more likely to adopt good oral health practices than are those with low perceived benefits and high perceived barriers, respectively [34, 36, 37]. However, our study revealed that individuals with higher perceived susceptibility were more likely to consume sweet foods daily and visit the dentist in the preceding 6 months, which contradicts the findings of previous studies [38, 39]. This discrepancy may be attributable to the strong association between parents’ perceived susceptibility and their child’s dental caries in our study. We suggest that when parents perceive a high risk of dental caries in their child, they are more likely to seek dental care. In addition, severe dental caries may lead to difficulties in normal eating, prompting parents to provide substitute foods, such as sweets.

Our study demonstrated a strong correlation between parents’ self-efficacy and positive oral health behaviors in their children. However, we did not find a similarly strong correlation between parents’ self-efficacy and children’s negative oral health behaviors. Self-efficacy, as originally conceptualized by Bandura, has been widely recognized as a key determinant of preventive health behaviors [15, 40]. A systematic review demonstrated a significant association between parental self-efficacy and preschoolers’ oral health behaviors [10]. In our study, children whose parents had higher self-efficacy were more likely to engage in positive oral health behaviors, such as brushing teeth twice daily, brushing teeth with parental assistance, and receiving a dental examination within the preceding 6 months. By contrast, children whose parents had lower self-efficacy were more prone to negative oral health behaviors, such as daily consumption of sugary foods. Our findings suggest that parental self-efficacy is a strong predictor of preschoolers’ positive oral health behaviors. Positive oral health behaviors—such as brushing teeth twice daily, brushing teeth with parental assistance, controlling sugar intake, and having regular dental examinations—can help prevent dental caries in children [17, 32]. Preschools and teachers should develop oral health education interventions that enhance parents’ perceptions of the benefits of oral care and their self-efficacy while addressing perceived barriers. This approach can promote positive oral health behaviors and improve management children’s of sugar intake.

Children aged 3 to 4 years exhibited the poorest behaviors in terms of brushing teeth twice daily and consuming sweet foods daily, whereas the behavior of brushing teeth with parental assistance was least frequently observed in children aged 5 to 6 years. In addition, our results indicated that the risk of dental caries increased with age, consistent with the findings of a study conducted in Shandong Province, which identified age as a crucial risk factor for dental caries in children [41]. Our study revealed a cumulative increase in the risk of dental caries and a decrease in OHRQoL as preschool children grew older. This suggests the need for age-specific interventions targeting various oral health behaviors. Furthermore, parental influence was found to be a crucial factor associated with children’s oral health and OHRQoL. Although the prevalence of dental caries was higher among fathers than among mothers, mothers reported lower levels of OHRQoL. This finding may be attributable to mothers typically being the primary caregivers for children (81.6%), leading to stronger oral health behavior–health outcome correlations for mothers and their children than for fathers and their children [9]. Thus, children’s oral health status may have a strong impact on mothers’ OHRQoL, and mothers often play a more effective role in improving their children’s OHRQoL than do fathers [14]. These results suggest that parental support programs should specifically empower mothers with age-appropriate oral health care skills so they can help their children adopt positive oral health behaviors. Such efforts can improve children’s oral health and thus their OHRQoL.

Despite our study being a large and representative survey, it has some limitations that should be acknowledged. First, as a cross-sectional analysis, our study discovered associations between the HBM constructs, children’s oral health behaviors, dental caries, and OHRQoL but could not establish causal relationships. Future oral health intervention studies conducted in preschool settings are needed to confirm causality among these factors. Second, although we used well-recognized scales with high reliability and validity in this study, the reliance on a self-reported questionnaire may have introduced information bias, particularly in behavioral data. Third, the assessment of children’s dental caries was based on parental self-reports, which may have negatively affected the validity of the findings. To minimize information bias, the researchers advised the parents to integrate objective data from recent dental examinations made by their child’s kindergarten and doctor-made diagnoses, which are received by parents on their mobile phone.

## Conclusion

The present study demonstrated that the constructs of the HBM are associated with children’s oral health behaviors, dental caries, and OHRQoL. In particular, parental oral health self-efficacy is the most crucial factor affecting children’s oral health behaviors.

## Electronic supplementary material

Below is the link to the electronic supplementary material.

Supplementary Material 1

## Data Availability

The datasets used and analyzed in the current study are available from the corresponding author on reasonable request.

## Abbreviations

HBM: Health Belief Model
ECC: Early Childhood Caries
OHRQoL: Oral Health–Related Quality of Life
AOR: Adjusted Odds Ratio
ECCPS: Early Childhood Caries Perceptions Scale
ECOHIS: Early Childhood Oral Health Impact Scale
CIS: Child Impact Section
FIS: Family Impact Section
