# Supplementary material for: Parental health belief model constructs associated with oral health behaviors, dental caries, and quality of life among preschool children in China: a cross-sectional study
Source: BMC Oral Health. 2024 Dec 18;24:1497. doi: 10.1186/s12903-024-05290-7 (PMC11657512; doi:10.1186/s12903-024-05290-7)
Supplement: Supplementary file 1 — Supplementary Material 1 [file 12903_2024_5290_MOESM1_ESM.docx]

| **Table S-1** Variables of Oral Health-Related Quality of Life | |
| --- | --- |
| Variables | Questions |
| Child impact | |
| 1. | How often has your child had pain in the teeth, mouth or jaws？ |
| 2. | How often has your child had difficulty drinking hot or cold beverages because of dental problems or dental treatments? |
| 3. | How often has your child had difficulty eating some foods because of dental problems or dental treatments? |
| 4. | How often has your child had difficulty pronouncing any words because of dental problems or dental treatments? |
| 5. | How often has your child missed pre-school, daycare or school because of dental problems or dental treatments? |
| 6. | How often has your child had trouble sleeping because of dental problems or dental treatments? |
| 7. | How often has your child been irritable or frustrated because of dental problems or dental treatments? |
| 8. | How often has your child avoided smiling or laughing of dental problems or dental treatments? |
| 9. | How often has your child avoided talking of dental problems or dental treatments? |
| Family impact | |
| 1. | How often have you or another family member been upset because of your child’s dental problems or dental treatments? |
| 2. | How often have you or another family member felt guilty because of your child’s dental problems or dental treatments? |
| 3. | How often have you or another family member taken time off from work because of your child’s dental problems or dental treatments? |
| 4. | How often has your child had dental problems or dental treatments that had a financial impact on your family? |
|  |  |

| **Table S-2.** Variables of Child’s Oral Health Behaviors | | | |
| --- | --- | --- | --- |
| Variables | Questions | | Response set |
| Brushing teeth twice daily | 1. | In the past month, did your child brush his or her teeth every morning and evening? | Yes, I did  No, I did not. |
| Brushing teeth with parental assistance | 2. | In the past month, did you help your child brush his or her teeth? | Yes, I did  No, I did not. |
| Consumption of sweet foods daily | 3. | In the past month, did your child consume sweet foods every day? | Yes, I did  No, I did not. |
| Recent dental examination | 4. | In the past six months, did your child visit the dentist for a dental examination? | Yes, I did  No, I did not. |

| **Table S-3.** Variables of Parental Oral Health Knowledge | |
| --- | --- |
| Variables | Questions |
| Dental knowledge | |
| 1. | Deciduous teeth will are replaced later and have no impact on on permanent teeth. |
| 2. | Untreated tooth decay in children can lead to uneven chewing and improper facial development. |
| 3. | Tooth decay can lead to systemic symptoms in children, such as toothache, facial swelling, and even high fever. |
| Dietary habits | |
| 1. | Providing children with excessive sweet foods or carbonated drinks often has a detrimental impact on their teeth. |
| 2. | Sleeping immediately after consuming milk does not adversely affect dental health. |
| 3. | Gargling after meals can help prevent cavities. |
| Oral hygiene tools | |
| 1. | Children’s toothbrushes should be replaced every six months. |
| 2. | When children plug teeth, parents can utilize dental floss or dental floss sticks to assist them in cleaning those spaces. |
| 3. | Using more toothpaste does not necessarily equate to better cleaning for a child’s teeth. |
| Tooth brushing routines | |
| 1. | It is challenging for young children to effectively brush their teeth, and parental assistance and supervision are needed. |
| 2. | Brushing teeth once daily can help prevent cavities in children. |
| 3. | Children should aim to brush their teeth for 2-3 minutes during each brushing session. |
| Dental visits | |
| 1. | Sealing cavities and grooves in the teeth of preschool children can prevent tooth decay. |
| 2. | Children should undergo oral health examination at least every six months. |
|  |  |

| **Table S-4.** Variables of Parental Oral Health Beliefs for ECC and ECC Prevention | |
| --- | --- |
| Variables | Questions |
| Susceptibility toward getting dental caries | |
| 1. | It is likely that my child will get tooth decay. |
| 2. | My child’s chances of getting cavities in the next few years are great. |
| 3. | Developing cavities is currently a possibility for my child. |
| 4. | I am concerned about my child’s likelihood of developing cavities in the near future. |
| Severity toward getting dental caries and its consequences | |
| 1. | Toothache can keep a child from sleeping through the night. |
| 2. | Child with cavities has bad breath. |
| 3. | Child with cavities is likely been teased by his/her friend. |
| 4. | Child with cavities makes parents spend money for dental treatment cost. |
| 5. | Child with cavities makes parents spend time for dental treatment. |
| Benefits toward practicing child’s oral health care | |
| 1. | Habitually child’s oral health cleaning will help my child avoid cavities. |
| 2. | Wiping child’s gum/tongue will help him/her avoid tooth decay. |
| 3. | Sipping water after every milk feeding will help my child avoid cavities. |
| 4. | Caries prevention will keep dental pain away from my child. |
| 5. | Caries prevention will help my child sleep through the night. |
| Barriers toward practice child’s oral health care | |
| 1. | A child’s toothbrush is too expensive for me to provide. |
| 2. | Brushing children’s teeth with a toothbrush is hard. |
| 3. | Practicing child’s oral cleaning would be painful for my child. |
| 4. | I don’t know how frequently to clean my child’s mouth. |
| 5. | My family members would not agree with forcing my child to cooperate in oral cleaning. |
|  |  |

| **Table S-5.** Variables of Parental Oral Health Self-efficacy | |
| --- | --- |
| Variables | Questions |
| Self-efficacy for brushing | |
| 1. | I am confident in finishing brushing my child’s teeth even if he or she feels sleepy. |
| 2. | I am confident in finishing brushing my child’s teeth every day. |
| 3. | I am confident in finishing brushing my child’s teeth even if he or she does not want me to do so. |
| 4. | I am confident in thoroughly finishing brushing my child’s teeth. |
| 5. | I am confident in finishing brushing my child’s teeth according to instructions. |
| 6. | I am confident in finishing brushing my child’s teeth according to instructions. |
| Self-efficacy for dietary habit | |
| 1. | I am confident I can resist buying snacks at the supermarket even if my child asks me to. |
| 2. | I am confident I can discourage lazy eating habits in my child. |
| 3. | I am confident I can refrain from offering sugary food to my child even if he or she becomes cranky or cries. |
| 4. | I am confident I can select snacks with low sugar content for my child. |
| 5. | I am confident I can encourage my child to eat various foods. |
| 6. | I am confident I can try to get my child into the habit of chewing thoroughly. |
| Self-efficacy for dentist consultations | |
| 1. | I am confident I can continue to take my child to a dentist for regular checkups after treatment is finished. |
| 2. | I am confident I can take my child to a dentist for regular checkups even if he or she is busy with after-school lessons. |
| 3. | I am confident I can take my child to a dentist for regular checkups even if I am busy with work or housework. |
| 4. | I am confident I can take my child to a dentist for regular checkups even if it means sacrificing my leisure time. |
